# Impacts of environmental factors on the aetiological diagnosis and disease severity of community-acquired pneumonia in China: a multicentre, hospital-based, observational study

**DOI:** 10.1017/S0950268824000700

**Published:** 2024-05-09

**Authors:** Yichunzi Zhang, Jiang Li, Chao Wu, Yan Xiao, Xinming Wang, Ying Wang, Lan Chen, Lili Ren, Jianwei Wang

**Affiliations:** 1 National Health Commission Key Laboratory of Systems Biology of Pathogens and Christophe Mérieux Laboratory, National Institute of Pathogen Biology, Chinese Academy of Medical Sciences and Peking Union Medical College, Beijing, China; 2 National Cancer Center/National Clinical Research Center for Cancer/Cancer Hospital, Chinese Academy of Medical Sciences and Peking Union Medical College, Beijing, China; 3 Key Laboratory of Respiratory Disease Pathogenomics, Chinese Academy of Medical Sciences and Peking Union Medical College, Beijing, China; 4 Key Laboratory of Pathogen Infection Prevention and Control (Ministry of Education), State Key Laboratory of Respiratory Health and Multimorbidity, National Institute of Pathogen Biology, Chinese Academy of Medical Sciences and Peking Union Medical College, Beijing, China

**Keywords:** aetiology, community-acquired pneumonia, disease severity, environmental factors, respiratory pathogens

## Abstract

Environmental exposures are known to be associated with pathogen transmission and immune impairment, but the association of exposures with aetiology and severity of community-acquired pneumonia (CAP) are unclear. A retrospective observational study was conducted at nine hospitals in eight provinces in China from 2014 to 2019. CAP patients were recruited according to inclusion criteria, and respiratory samples were screened for 33 respiratory pathogens using molecular test methods. Sociodemographic, environmental and clinical factors were used to analyze the association with pathogen detection and disease severity by logistic regression models combined with distributed lag nonlinear models. A total of 3323 CAP patients were included, with 709 (21.3%) having severe illness. 2064 (62.1%) patients were positive for at least one pathogen. More severe patients were found in positive group. After adjusting for confounders, particulate matter (PM) 2.5 and 8-h ozone (O_3_-8h) were significant association at specific lag periods with detection of influenza viruses and *Klebsiella pneumoniae* respectively. PM10 and carbon monoxide (CO) showed cumulative effect with severe CAP. Pollutants exposures, especially PM, O_3_-8h, and CO should be considered in pathogen detection and severity of CAP to improve the clinical aetiological and disease severity diagnosis.

## Graphical abstract

**Figure:**
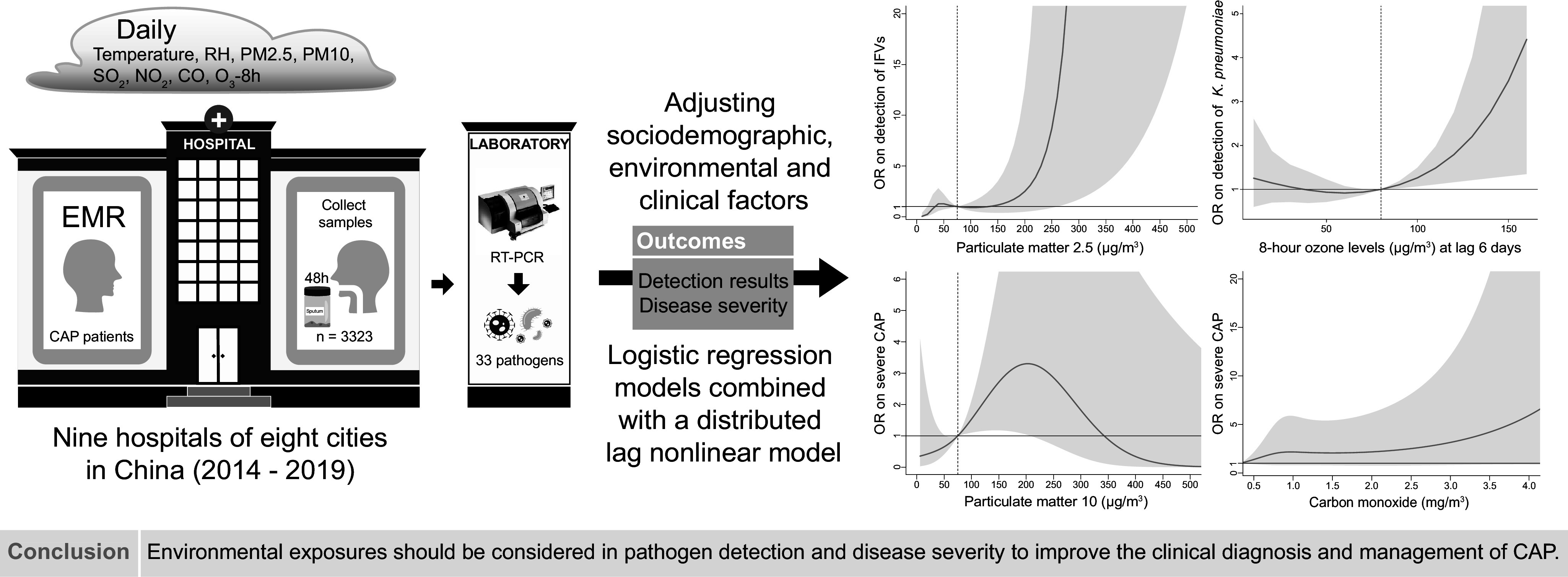

## Introduction

Community-acquired pneumonia (CAP) is one of the leading causes of the disease burden worldwide, representing a major global clinical and public health issue [1, 2]. The annual incidence of CAP is 1.07–7.03 cases per 1 000 adults [2, 3], and the annual incidence of severe pneumonia among adults ranges from 0.14 to 0.17 per 1 000 population [4]. An understanding of the aetiology of CAP can improve clinical treatment and vaccine and drug development, especially when molecular tests with high sensitivity are used [3, 5]. Previous studies have focused on the impact of environmental factors on the incidence or mortality associated with pneumonia; however, the effect of environmental factors on the pathogen detection rate and severity of CAP has still not been evaluated intensively.

Exposure to air pollution with fine particulate is associated with the increasing of mortality [6]. Ozone (O_3_) can impair small airway function, increasing the risk of small airway dysfunction [7]. In subtropical and temperate regions, the activity of respiratory syncytial virus is greater at lower temperatures and higher relative humidity (RH) [8]. Additionally, the incidence of CAP is higher among males [9, 10]. Disease severity is also associated with age, sex, and lifestyle [11, 12]. Current findings suggest that the effects of environmental factors and medical behaviours on the disease and aetiology of CAP should be considered intensively.

In this study, we explored the effect of environmental factors, including temperature, RH, and air pollutants, on aetiological detection and severity in CAP patients by adjusting sociodemographic variables and medical behaviours. Our findings provide insights to improve the understanding of environmental factors affecting the aetiology and severity of CAP.

## Materials and methods

### Study design and population

This cross-sectional study was designed according to the Strengthening the Reporting of Observational Studies in Epidemiology (STROBE) statement guideline (Supplementary Text S1). CAP and severe CAP (sCAP) were defined according to the 2007 Infectious Disease Society of America/American Thoracic Society CAP guideline [13]. CAP patients were recruited according to the criteria from 1 January 2014 to 31 December 2019, from nine hospitals located in eight cities, including Shenzhen, Fuzhou, Nanjing, Harbin, Changchun, Wuhan, Chengdu, and Xi’an, in China. Patients with immunosuppression or noninfectious pneumonia were excluded (Supplementary Table S1 and Supplementary Text S2).

### Procedures

Respiratory samples including sputum or bronchoalveolar lavage fluid were collected from each patient within 48 h after admission. Multiplex real-time PCR (Fast-Track Diagnostics, Junglinster Luxembourg) was used to screen for 33 respiratory pathogens [14] (Supplementary Text S3). All pathogen screening was completed by the central laboratory. Bacteria and fungi were defined as bacteria (fungus), and *Pneumocystis jiroveci* (*P. jirovecii*) was the only fungus detected in our study. Demographic, clinical information and pathogen screening results were collected from clinical records, including age, sex, body mass index (BMI), antibiotics using 5 days pre-admission (AP), time from symptom onset to admission (TFSOA), and the days between admission and sampling. Age was grouped by 5-year intervals [15]. Sex, BMI, and AP were coded as binary variables. A BMI ≥ 25 kg/m^2^ was considered overweight. The pneumonia severity index (PSI) score was extracted and used in the positive detection model. A PSI score ≥ 90 was considered sCAP [16].

Daily RH and temperature data were derived from environmental datasets provided by the China Meteorological Administration, and pollutants, including particulate matter (PM) 2.5, PM10, sulphur dioxide (SO_2_), nitrogen dioxide (NO_2_), 8-h O_3_ (O_3_-8h) levels and carbon monoxide (CO), at each geographical site of the sentinel hospital from the national urban air quality platform were provided by the China National Environmental Monitoring Centre. The air pollutant data before May 2014 were collected from the China air quality online monitoring and analysis platform. The emission standard concentrations of pollutants were 75 μg/m^3^, 150 μg/m^3^, 150 μg/m^3^, 80 μg/m^3^, 160 μg/m^3^, and 4 mg/m^3^ according to Ambient Air Quality Standards. Considering time differences in the impact of environmental variables on outcomes, the severity and pathogen detection were respectively matched with admission and sampling time. Based on the cumulative effect of environmental factors on lung function, multiple-day lags (from lag 0–1 to lag 0–6) were matched to the environmental variables, while only temperature [17, 18] was matched to a 3-day moving average (lag 0–2 days) [19].

### Outcome measures

The primary outcomes were defined as pathogen detection and disease severity. The effect of environmental variables on pathogen detection and severity was analyzed. Specific pathogens with high frequency were involved, including *Mycoplasma pneumoniae* (*M. pneumoniae*), *Haemophilus influenzae* (*H. influenzae*), *Klebsiella pneumoniae* (*K. pneumoniae*), *Streptococcus pneumoniae* (*S. pneumoniae*), influenza viruses (IFVs), and human rhinovirus (HRV).

### Statistical analysis

With a maximum of 18 variables with a minimum of 14–20 events per variable, the events per variable were used to estimate the sample size [20]. The *χ* ^2^ test, Mann–Whitney *U* test and Kruskal–Wallis *H* test were used to evaluate bivariate association in the dataset with lag 0–6. Phi correlation coefficients were used to assess coinfection between pathogens. To explore the relationship between air pollutants and outcomes, we established both logistic regression models and logistic regression models combined with the distributed lag nonlinear model (DLNM) for pathogen detection results and severity of CAP respectively, reporting adjusted odds ratios (ORs) and 95% confidence intervals (CIs). Demographic and environmental factors, area, and admission time were adjusted for in logistic regression models on the basis of the significance of bivariate association and previous knowledge (Supplementary Tables S2 and S3). Estimated changes in tested pathogens and pneumonia severity were evaluated given a 10-μg/m^3^ increment in PM2.5, PM10, SO_2_, NO_2_, and O_3_-8h exposure [21], given a 1-mg/m^3^ increment in CO exposure [22], given a 10% increment in RH exposure [23], and given a 1°C increment in temperature. While variables and models of DLNM were shown in Supplementary Text S4.

Multicollinearity was examined using the variance inflation factor (VIF) [24]. The examination results of all included variables were under 10 by VIF (Supplementary Table S4). We further considered the possible collinearity or interaction between pollutants and applied the Bayesian kernel machine regression (BKMR) model, which allowed us to evaluate the effect of combined exposure. The model adjusted above confounding factors, including sociodemographic variables, medical behaviours, temperature and RH, and ran up to 10000 iterations using the Markov chain Monte Carlo (MCMC) algorithm.

The missing rates of age, sex, and BMI were lower than 5%, except for age, which had a rate of 11.9% (Supplementary Table S5). Multiple imputation with MCMC methods combined with Rubin’s rules was used to treat the missing data, assumed to be missing at random, supposing that the missing data were dependent on the observed variables. The estimated effect in the logistic regression models was pooled. The estimated effects in the DLNM and BKMR were from the imputation dataset according to the minimized value of the Akaike information criterion.

We conducted a case-crossover study design as sensitivity analysis to assess the robustness of the study. Each patient’s date of admission (event day) was matched with the days before event day as referent days in the same area, year, and day of week. Each patient was guaranteed at least 3 referent days. Since the case-crossover study design is a self-matched study, both observed and unobserved time-invariant confounding are controlled for by design. After adjusting other environmental parameters, Conditional logistic regression models were used to estimate adjusted ORs (95% CIs). All statistical tests were two-sided, and a *P* value less than 0.05 was considered statistically significant. All analyses were conducted using SPSS (version 22, IBM SPSS Statistics for Windows, Armonk, NY) and R (version 4.2.3, R Core Team, Vienna, Austria).

## Results

**Figure 1.**
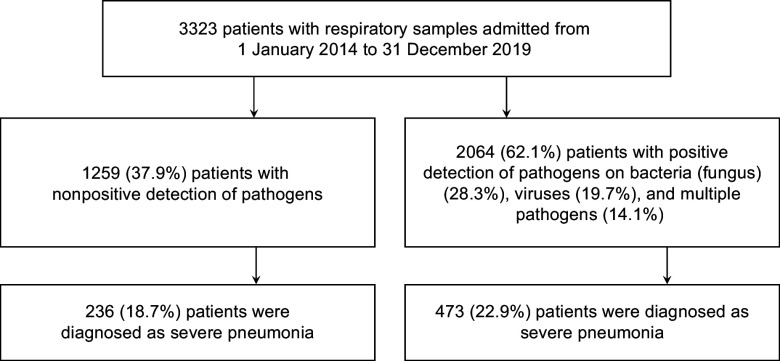
Flowchart of including patients in the study.

**Figure 2.**
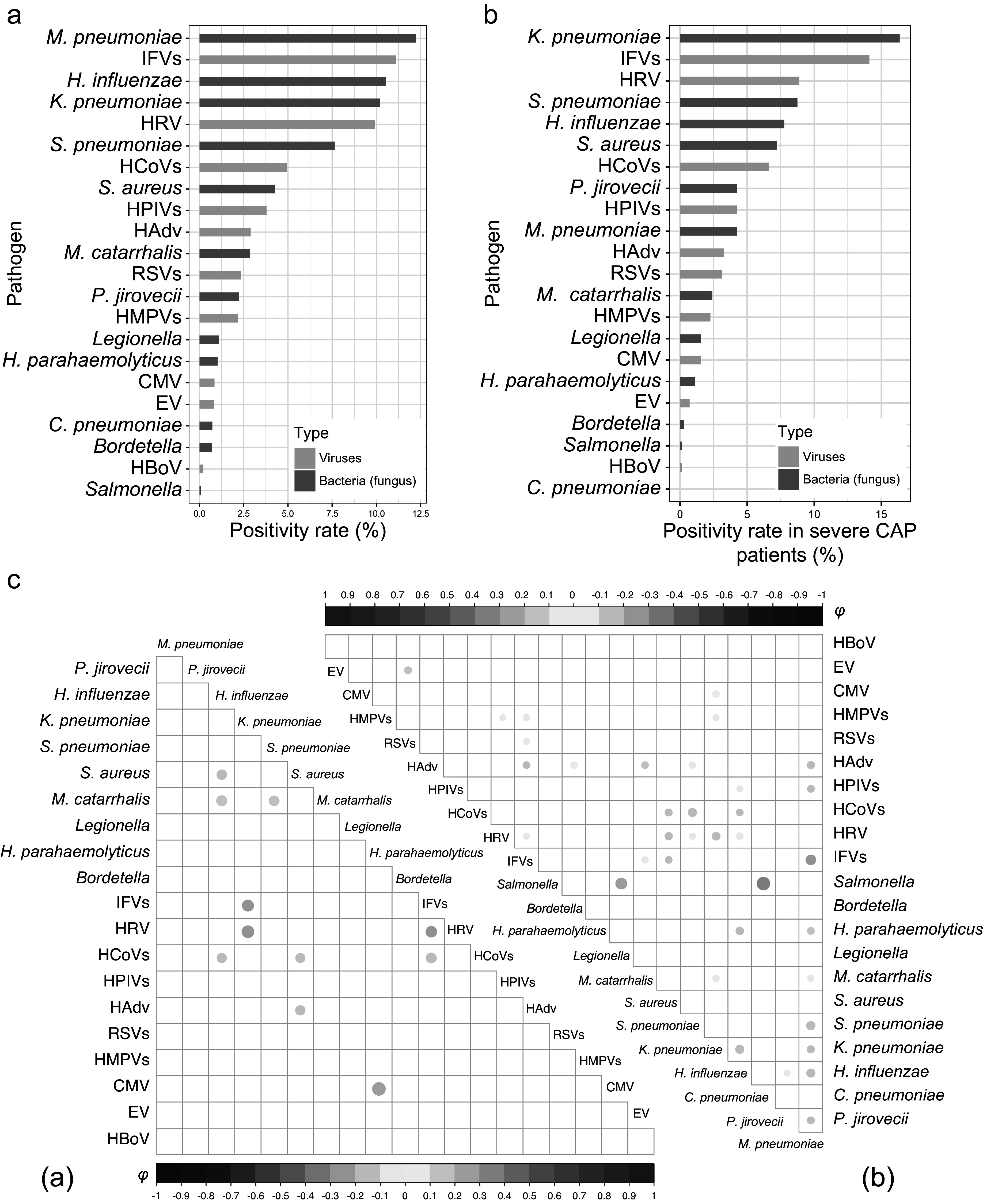
Pathogen detection in patients with community-acquired pneumonia (CAP). (a) Proportion of detected pathogens in tested CAP patients. (b) Pathogen positivity rate among severe CAP patients. (c) Pathogen codetections in severe (a) and nonsevere (b) CAP patients analyzed by Phi correlation coefficients. *C. pneumoniae, Chlamydia pneumoniae*; CMV, cytomegalovirus; EV, enterovirus; *H. influenzae, Haemophilus influenzae*; *H. parahaemolyticus, Haemophilus parahaemolyticus*; HAdv, human adenovirus; HBoV, human bocavirus; HCoVs, human coronaviruses; HMPVs, human metapneumoviruses; HPIVs, human parainfluenza viruses; HRV, human rhinovirus; IFVs, influenza viruses; *K. pneumoniae, Klebsiella pneumoniae*; *M. catarrhalis, Moraxella catarrhalis*; *M. pneumoniae, Mycoplasma pneumoniae*; *P. jirovecii, Pneumocystis jiroveci*; RSVs, respiratory syncytial viruses; *S. aureus, Staphylococcus aureus*; *S. pneumoniae, Streptococcus pneumoniae.*

A total of 3323 CAP patients with pathogen testing results were enrolled, with 709 (21.3%) sCAP patients (Figure 1). A total of 1936 (58.3%) patients were male. The median age of the enrolled patients was 58 years (interquartile range (IQR): 40–69). A total of 550 (16.6%) patients were overweight. At least one pathogen was detected in 2064 (62.1%) patients, with 942 (28.3%) positive for bacterial (fungal) infections, 653 (19.7%) positive for viral infections, and 469 (14.1%) positive for multiple pathogens. The distribution of pathogen detection results showed that the aetiology of CAP was still mainly bacterial (fungal), followed by viral and due to multiple pathogens (Supplementary Table S6). Among all the detected pathogens, *M. pneumoniae* was the most frequently detected pathogen, accounting for 12.2% (*n* = 407), followed by IFVs (11.1%), *H. influenzae* (10.5%), *K. pneumoniae* (10.2%), HRV (9.9%), *S. pneumoniae* (7.6%), human coronaviruses (HCoVs, 4.9%), *Staphylococcus aureus* (*S. aureus*, 4.3%), human parainfluenza viruses (3.8%), human adenovirus (2.9%), *Moraxella catarrhalis* (*M. catarrhalis*, 2.9%), respiratory syncytial viruses (RSVs, 2.3%), *P. jirovecii* (2.2%), human metapneumoviruses (2.2%), *Legionella* spp. (1.1%) and *Haemophilus parahaemolyticus* (*H. parahaemolyticus*, 1.0%), whereas the other pathogens had a positive detection rate lower than 1% (Figure 2a). The demographic characteristics, as well as the pathogen detection results of the study population, are shown in Table 1. A total of 782 (23.5%) patients reported AP. The median TFSOA was 7 days (IQR: 3–10) (Table 1). The concentrations of lag 0–6 days for temperature, RH, and exposure pollutants in the studied population are summarized in Supplementary Table S7. The detailed characteristics of the study population are further shown after grouping by pathogen detection results and disease severity (Supplementary Tables S2 and S3).

**Table 1.** Clinical and demographic characteristics of community-acquired pneumonia patients

Variables	Participants (*n* = 3323)
Age, years; median (IQR)	58 (29)
Sex	
Female; *n* (%)	1251 (37.6)
Male; *n* (%)	1936 (58.3)
BMI, kg/m^2^	
<25; *n* (%)	2609 (78.5)
≥25; *n* (%)	550 (16.6)
sCAP	
No; *n* (%)	2614 (78.7)
Yes; *n* (%)	709 (21.3)
Pathogen	
Nonpositive detection; *n* (%)	1259 (37.9)
Bacteria (fungus); *n* (%)	942 (28.3)
Viruses; *n* (%)	653 (19.7)
Multiple pathogens; *n* (%)	469 (14.1)
TFSOA, days; median (IQR)	7 (7)
AP	
No; *n* (%)	2541 (76.5)
Yes; *n* (%)	782 (23.5)
PSI score	
<90; *n* (%)	2720 (81.9)
≥90; *n* (%)	603 (18.1)

Except sex and BMI, not all percentages add up to 100% due to rounding. AP, antibiotics pre-admission; BMI, body mass index; IQR, interquartile range; PSI, pneumonia severity index; sCAP, severe community-acquired pneumonia; TFSOA, time from symptom onset to admission.

Compared with nonpositive patients (18.7%, 236 of 1259), patients with positive pathogen detection (22.9%, 473 of 2064, adjusted OR = 1.40, 95% CI: 1.16–1.68) had a higher sCAP rate (Supplementary Table S8). Specifically, *K. pneumoniae* (16.4%), IFVs (14.1%), *S. aureus* (7.2%), HCoVs (6.6%), *P. jirovecii* (4.2%) and cytomegalovirus (CMV, 1.6%) were more frequent in sCAP patients than in nonsevere CAP patients (*P* < 0.02, Figure 2b). *M. pneumoniae* was negatively associated with sCAP (adjusted OR = 0.45, 95% CI: 0.27–0.75). The median age of patients with sCAP (63, IQR: 49–74; adjusted OR = 1.09, 95% CI: 1.07–1.12) was older than that of patients with nonsevere CAP (56, IQR: 37–68). In elderly patients, *K. pneumoniae* (adjusted OR = 1.06, 95% CI: 1.02–1.10) and IFVs (adjusted OR = 1.04, 95% CI: 1.00–1.08) were found in high frequency, but *M. pneumoniae* was less detected (adjusted OR = 0.83, 95% CI: 0.80–0.86) (Supplementary Table S9). The proportion of sCAP was higher in males (25.6%, 496 of 1936) than in females (15.3%, 192 of 1251) (adjusted OR = 1.83, 95% CI: 1.51–2.21). *K. pneumoniae* (adjusted OR = 1.37, 95% CI: 1.06–1.77) and *S. pneumoniae* (adjusted OR = 1.55, 95% CI: 1.16–2.08) were found in high frequency in male patients. As of codetection, *M. catarrhalis* specifically co-detected with *H. influenzae* (*φ* = 0.19, *P* = 0.02) and *S. pneumoniae* (*φ* = 0.17, *P* = 0.03) in sCAP patients, while *H. parahaemolyticus* was specifically co-detected with CMV (*φ* = 0.29, *P* = 0.02, Figure 2c).

**Figure 3.**
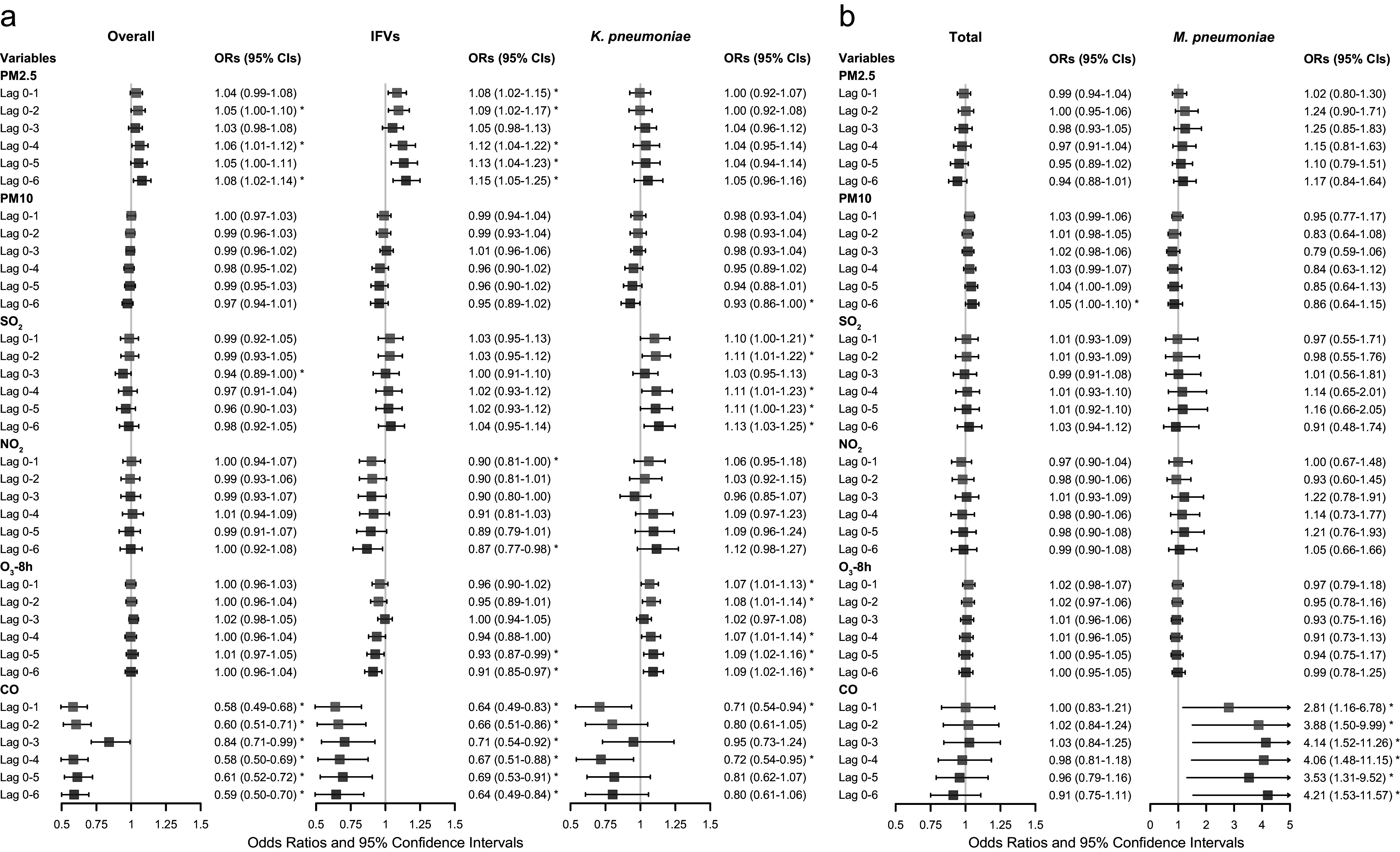
Adjusted ORs (95% CIs) for pathogen detection and severe community-acquired pneumonia (CAP) with increased environmental concentrations according to the logistic regression models. (a) Association of environmental parameters with overall pathogen detection, detection of influenza viruses and *Klebsiella pneumoniae*, adjusted for age, sex, BMI, temperature, RH, PM2.5, PM10, SO_2_, NO_2_, O_3_-8h, CO, AP, TFSOA, pneumonia severity index score, area, and admission time. (b) Association of environmental parameters with severe CAP in total patients and patients detected with *Mycoplasma pneumoniae*, adjusted for age, sex, BMI, temperature, RH, PM2.5, PM10, SO_2_, NO_2_, O_3_-8h, CO, AP, TFSOA, area, and admission time. Pathogen detection was extra adjusted in model of total patients. AP, antibiotics pre-admission; BMI, body mass index; CO, carbon monoxide; NO_2_, nitrogen dioxide; O_3_-8h, 8-h ozone levels; OR, odds ratio; PM, particulate matter; RH, relative humidity; SO_2_, sulphur dioxide; TFSOA, time from symptom onset to admission.

**Figure 4.**
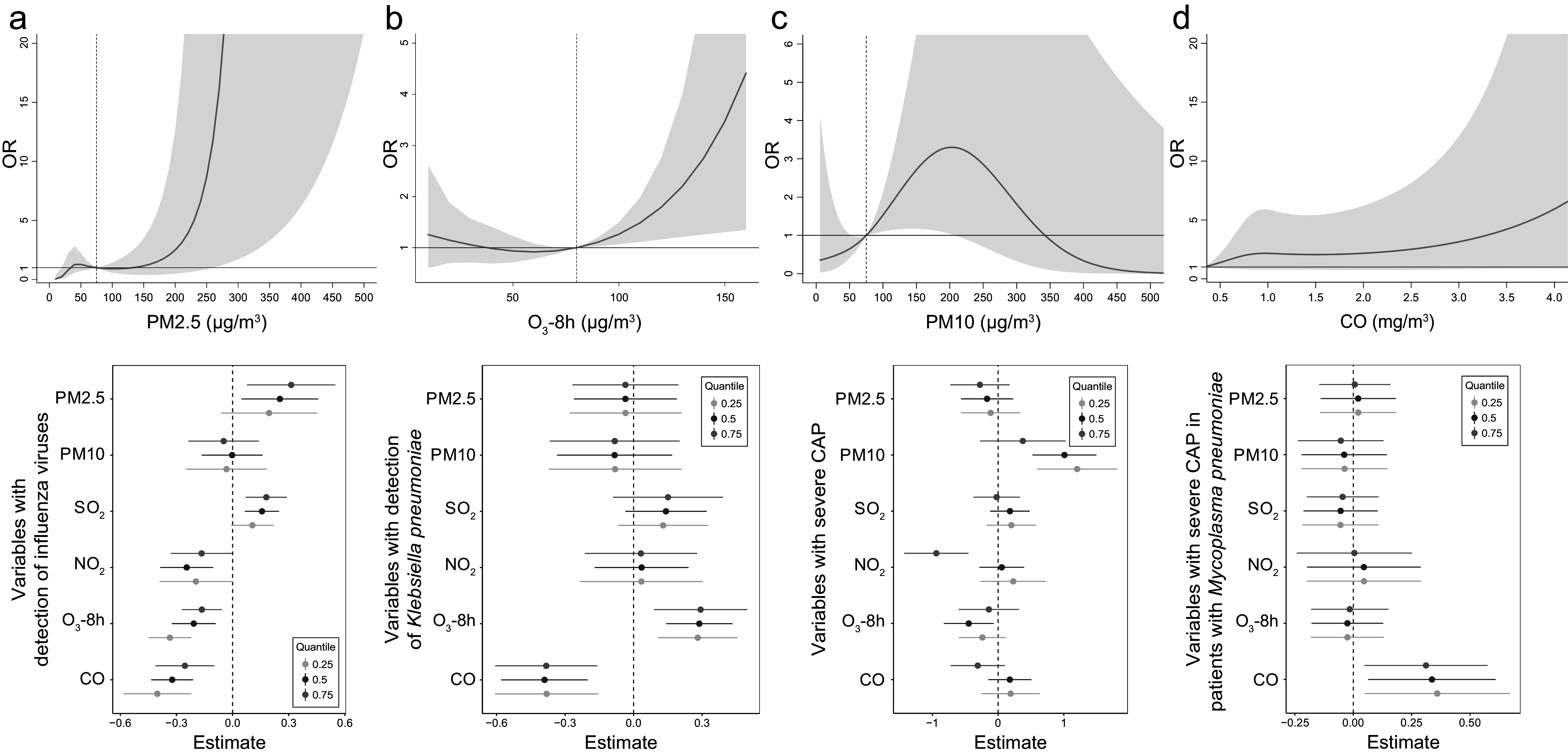
Significant association of specific environmental variables with the detection of specific pathogens and severe community-acquired pneumonia (CAP). (a) For the association of PM2.5 on detection of influenza viruses, exposure-response curve according to distributed lag nonlinear model (DLNM), and single-exposure effects according to Bayesian kernel machine regression (BKMR). The dashed line in DLNM is 75 μg/m^3^, representing the concentration of emission standard. (b) Exposure-response curve at lag 6 days and single-exposure effects for the association of O_3_-8h on detection of *Klebsiella pneumoniae.* The dashed line is 80 μg/m^3^, representing half of emission standard. (c) In total CAP patients, exposure-response curve and single-exposure effects for association of PM10 on severe CAP. The dashed line is 75 μg/m^3^, representing half of emission standard. (d) For the association of CO on severe CAP, exposure-response curve in total CAP patients and single-exposure effects in CAP patients detected with *Mycoplasma pneumoniae.* The compared concentration of CO is the minimum. Effects from BKMR were defined as the change in the response associated with a change in a particular exposure from its 25th to its 75th percentile, where all of the other exposures are fixed at a specific quantile (0.25, 0.50, or 0.75). CO, carbon monoxide; NO_2_, nitrogen dioxide; O_3_-8h, 8-h ozone levels; OR, odds ratio; PM, particulate matter; SO_2_, sulphur dioxide.

The environmental parameters PM2.5 and O_3_-8h were significantly associated with pathogens positive detections. As of PM2.5, each 10-μg/m^3^ increment in PM2.5 was significantly associated with positive detections with the adjusted OR of 1.08 (95% CI: 1.02–1.14), and with the detection of IFVs at lag 0–6 days (adjusted OR = 1.15, 95% CI: 1.05–1.25, Figure 3a). The detection of IFVs in PM2.5 of lagged 0–6 days at 260 μg/m^3^ was significantly more common than that in PM2.5 at emission standard (75 μg/m^3^, adjusted OR = 11.76, 95% CI: 1.00–137.85) analyzed by using DLNM. The result of BKMR showed that PM2.5 affected the detection of IFVs independently (Figure 4a). The increment of PM2.5 was also significant association with detection of *H. influenzae* with the adjusted OR = 1.13, 95% CI: 1.02–1.24 (Supplementary Figure S1). DLNM showed that PM2.5 at lag 0 day was significantly associated with the detection of *H. influenzae* when concentration was six times higher than emission standard. However, the exposure of PM2.5 showed no significant effect on the detection of *H. influenzae* when analyzed using BKMR (Supplementary Figure S2a). There was also a positive association between increased O_3_ concentration and the detection of *K. pneumoniae* (adjusted OR = 1.09, 95% CI: 1.02–1.16, Figure 3a) at lag period of 0–6 days. A significant association between O_3_-8h and the detection of *K. pneumoniae* was also shown in the DLNM at lag 6 days when the concentration of O_3_-8h was double the half of emission standard (80 μg/m^3^, adjusted OR = 4.41, 95% CI: 1.35–14.44). O_3_-8h affected the detection of *K. pneumoniae* independently according to BKMR (Figure 4b).

Of other environmental factors, SO_2_ showed significant association with positive-detection of *K. pneumoniae* (adjusted OR = 1.13, 95% CI: 1.03–1.25, Figure 3a), and positive effect presented at lag 4 days when the concentration of SO_2_ was more than half of emission standard according to the analysis of DLNM (Supplementary Figure S2b). However, SO_2_ showed no significant effect on the detection of *K. pneumoniae* according to BKMR (Figure 4b). We also found each 10-μg/m^3^ increment in NO_2_ was significantly associated with HRV (adjusted OR = 1.21, 95% CI: 1.07–1.37, Supplementary Figure S1) at lag 0–5 days. While the effect was not significant in DLNM (Supplementary Figure S2b). Apart from pollutants, RH showed association with positive detection (adjusted OR = 1.09, 95% CI: 1.03–1.16) and viral detection (adjusted OR = 1.18, 95% CI: 1.09–1.28) at lag 0–5 days, and compared with RH at 50%, cumulative effect of lag 0–5 days in RH at 80% was 2.25 (95% CI: 1.07–4.71, Supplementary Figure S2c).

PM10 and CO were significantly associated with sCAP. There was a significant association between PM10 and the sCAP at lag 0–6 days (adjusted OR = 1.05, 95% CI: 1.00–1.10, Figure 3b). Compared with half of emission standard, the cumulative effect at lag 0–6 days was 2.71 (95% CI: 1.18–6.26, Figure 4c) when the concentration of PM10 was at emission standard (150 μg/m^3^). In addition, a 1-mg/m^3^ increment in CO at lag 0–6 days was significantly associated with sCAP in patients detected with *M. pneumoniae* (adjusted OR = 4.21, 95% CI: 1.53–11.57, Figure 3b). PM10 independently affected sCAP in all patients, and CO independently affected sCAP positive on *M. pneumoniae* analyzed by using BKMR (Figure 4c,d). While a negative association was found between CO and the detection of pathogen (Figure 3a and Supplementary Figure S3). For other association with sCAP, it was observed that PM10 (adjusted OR = 1.39, 95% CI: 1.14–1.68) and SO_2_ (adjusted OR = 2.05, 95% CI: 1.32–3.16, Supplementary Figure S4) were significantly associated with sCAP in patients detected with HRV, but the effects of them seemed to be dependent (Supplementary Figure S2d).

Our sensitivity analysis for more stringent case-crossover study design illustrated a trend of robustness in our results. After adjusting confounding environmental parameters, it showed that the exposure of PM2.5 was associated with the detection of IFVs (adjusted OR = 1.02, 95% CI: 1.00–1.04), and the exposure of O_3_-8h was associated with detection of *K. pneumoniae* (adjusted OR = 1.04, 95% CI: 1.02–1.06). While the association between RH and detection of viruses was not significant in case-crossover study design. PM10 showed a significant association with sCAP (adjusted OR = 1.01, 95% CI: 1.00–1.01), and CO showed the association with sCAP (adjusted OR = 3.24, 95% CI: 1.08–9.79) in patients detected with *M. pneumoniae.* There was no significant association between other environmental parameters and outcomes in our study (Figure 3 and Supplementary Figures S1 and S3–S5).

Except for environmental factors, positive pathogen detection was also affected by the medical behaviours of patients, including TFSOA and AP (Supplementary Table S9). TFSOA was negatively associated with pathogen detection. Negative associations between TFSOA and the detection of *M. pneumoniae*, *H. influenzae*, *S. pneumoniae*, and IFVs were observed. In addition, AP was positively associated with overall pathogen detection, especially with *M. pneumoniae* (adjusted OR = 1.75, 95% CI: 1.36–2.25) and IFVs (adjusted OR = 1.46, 95% CI: 1.13–1.88) detection.

## Discussion

We conducted a multicentre hospital-based observational study to investigate the association of environmental factors with the aetiological diagnosis and severity of CAP in China. We found that environmental parameters, especially PM2.5 and O_3_-8h, showed a significant association with positive detections of CAP. In particular, IFVs were detected mostly when patients were exposed to high concentrations of PM2.5. The increment of O_3_-8h more than 80 μg/m^3^ was positively associated with the detection of *K. pneumoniae*, especially when the exposure to O_3_-8h occurred on the last 6 days. We also found that PM10 and CO showed a significant association with sCAP. Compared with a PM10 of 75 μg/m^3^, the exposure of double concentration showed the greater positive association with sCAP. And as the increment of CO, there was positive association with sCAP in patients detected with *M. pneumoniae*, while negative association with the detection of pathogens in whole patients. In addition, a long TFSOA was negatively associated with overall pathogens, especially *M. pneumoniae*, *H. influenzae*, *S. pneumoniae*, and IFVs according to this study.

The associations of air pollutants with CAP hospitalizations and mortality have been described in detail [25, 26]. A previous study described the association of aetiological detection of CAP with weather variables and pollutants according to the correlation coefficient, and they reported that increased SO_2_ levels led to an increased rate of detection according to models adjusted for time trends, RH, and temperature only [27]. We used more rigorous inclusion criteria for pneumonia cases and extracted detailed clinical data to define severe pneumonia. After adjusting for other environmental parameters, demographics, behaviours and severity, the effects of PM2.5 and O_3_-8h on the detection of CAP were shown in a larger sample size, and the effects of PM10 and CO on sCAP were shown in our study. The DLNM enabled us to elucidate the multiple-day effects of a single day of exposure, and the BKMR benefited the study of single-exposure in environmental parameters.

Consistent with other studies, male sex and old age were high-risk factors for CAP [11]. A study in Utah with a larger sample size reported that PM2.5 and O_3_ showed a positive association with sCAP after stratification by age but without adjusting for sex or detected pathogens [28]. However, PM2.5 and O_3_ were positively associated with the detection of pathogens but not severity in our study. It is necessary to consider the effect of environmental factors on the aetiological diagnosis of CAP when studying severity.

Environmental factors can affect host susceptibility by modulating airway defence mechanisms and affecting the viability and transmission of pathogens. PM10 and PM2.5 aggravate the immune response by entering the human respiratory tract. For example, PM2.5 can modulate the innate immune system of the respiratory tract through mechanisms such as inflammation mediated by alveolar macrophages, recruitment of neutrophils, disruption of barrier defences, and upregulation of receptors and molecules involved in the procedure of pathogens invasion, making the inhalation of airborne transmission of respiratory viruses possible [29, 30]. This might explain our observation of an association with IFVs and an increase in PM2.5, and the observation of an association with sCAP and an increase in PM10. A population-based study described a significant association of PM2.5 concentration with the incidence of influenza-like illness [31]. Both the cumulative effect of PM2.5 on the detection of IFVs and the cumulative effect of PM10 on sCAP could last 6 days in our study.

O_3_ is usually considered an antimicrobial agent. Low-dose gaseous ozone was reported to inhibit the growth of clinical isolates of *K. pneumoniae* [32]. It has been reported that tropospheric O_3_ could cause peroxidation of lipids in the nasal and airway lining liquid and epithelial cell membranes, leading to epithelial cell damage and subsequent sterile inflammation [33]. O_3_ was an independent risk factor for respiratory bacterial and multidrug-resistant bacteria infections, as reported previously [34]. Our study reported a positive effect of O_3_-8h on *K. pneumoniae* in the study population, which has rarely been reported in previous studies and might be explained by *K. pneumoniae* disrupting the mucosal barrier at the colonization site and allowing the pathogen to escape the colonization site to establish an infection, or directly allowing the pathogen to enter the body [35]. The positive effect of O_3_-8h on *K. pneumoniae* could lag 6 days when the O_3_-8h level was over half of the emission standard according to our study.

The detection of pathogens was significantly negative association with increases in CO levels, although during our study the concentration of CO never exceeded the threshold range defined by pollutant emissions. However, a positive association with increase in CO levels on sCAP was observed in patients with *M. pneumoniae.* As an exogenous toxic gas [22], inhalation through the respiratory tract is the main way ambient CO enters the human body. Circulating CO exerts its toxic effect by binding to heme and altering the function and metabolism of heme protein, which may lead to tissue hypoxia damage and trigger inflammatory and stress responses [36]. Our study suggested the underlying immune perturbations by the exposure of CO, even less than emission standard, on potential CAP patients. The reported study also showed that CO, at low concentrations, was also considered an antiapoptotic, antiproliferative and anti-inflammatory factor [37]. This might explain the insignificant effect of CO on sCAP in all patients. In addition, RH, ranging from 20 to 100%, was positively associated with the positive-detection of viruses, especially the RH at 80%, which might be explained by its effect on infectious droplets in respiratory viruses. While this effect was not significant in the case-crossover study.

The lack of an association might be explained by two main points. First, different pathogens showed different affected traits according to the variant effects of environmental parameters on specific pathogens in the above study, which might explain the different effects between pathogens and specific pathogens. Second, an analysis of the effects of environmental parameters on other specific pathogens, including HCoVs, *S. aureus*, RSVs, *P. jirovecii*, CMV, and so on, was not conducted owing to the small number of patients with these pathogens.

Additionally, AP was positively associated with the detection of *M. pneumoniae* and IFVs in our study. By weakening the competitive exclusion of pathogens and inducing the emergence of antibiotic-resistant bacterial strains, the initial use of unnecessarily broad-spectrum antibiotics is associated with increased in-hospital mortality and might be a risk factor for fulminant *M. pneumoniae* pneumonia and lung vulnerability to IFVs [38, 39].

Early and accurate diagnosis of CAP is crucial to initiate targeted therapy [40]. This fact requires strengthening the detection of high-frequency and high-risk pathogens in patients and improving the relevance of diagnosis and treatment plans. Pathogen detection and severity of CAP were affected by environmental factors according to our study. The results suggest that some environmental factors affecting the lungs might directly perturb regional immunity. Thus, the effect might involve impairing airway defence mechanisms, such as with PM2.5, PM10, O_3_, and CO, and increasing the transmission of pathogens, such as with PM2.5 and RH. Demographic variables, PM2.5, PM10, O_3_, CO, AP, and TFSOA should be taken into consideration both in clinical pathogen detection and in potential CAP patient self-management.

Our study has several limitations. First, our dataset was hospital-based, and the patients were mostly located in areas with better socioeconomic development than average. Future population-based and experimental studies are necessary to discover the underlying mechanism. Second, respiratory pathogens showed different traits affected by environmental factors. *S. aureus*, HCoVs, *P. jirovecii*, and CMV were more highly detected in sCAP patients but were not intensively evaluated in this study owing to limited samples. Furthermore, there was an association between detection results and severity of CAP in an exploratory study. To precisely study the effect of environmental parameters on one of the outcomes, we adjusted the other one. While potential mediating effect should be fully evaluated in a larger sample size and a more precise study design. The effects of environmental parameters on other pathogens, and more complex association between factors can be furtherly estimated in a larger sample size.

## Conclusions

O_3_-8h, PM2.5, and TFSOA were associated with respiratory pathogen detection, especially the effect of PM2.5 on IFVs could last 6 days, the effect of O_3_-8h more than 80 μg/m^3^ on *K. pneumoniae* was at lag 6 days. PM10 and CO were significantly associated with sCAP in cumulative effect. Our findings have important implications for improving the understanding of environmental factors in the aetiological diagnosis and severity of CAP and improving health care.

## Supporting information

Zhang et al. supplementary material Zhang et al. supplementary material

## Data Availability

The dataset used and analyzed during this study is available from the corresponding author upon reasonable request.

## List of abbreviations

AP: antibiotics pre-admission
BMI: body mass index
CAP: community-acquired pneumonia
CI: confidence interval
CO: carbon monoxide
NO_2_: nitrogen dioxide
O_3_-8h: 8-h ozone
OR: odds ratio
PM: particulate matter
PSI: pneumonia severity index
RH: relative humidity
sCAP: severe community-acquired pneumonia
SO_2_: sulphur dioxide
TFSOA: time from symptom onset to admission

## Supplementary material

For supplementary material accompanying this paper visit http://doi.org/10.1017/S0950268824000700. click here to view supplementary material
