# Supplementary material for: Impacts of environmental factors on the aetiological diagnosis and disease severity of community-acquired pneumonia in China: a multicentre, hospital-based, observational study
Source: Epidemiol Infect. 2024 May 9;152:e80. doi: 10.1017/S0950268824000700 (PMC11131030; doi:10.1017/S0950268824000700)
Supplement: Zhang et al. supplementary material [file S0950268824000700sup001.zip › Zhang_Supplementary Material-R3 - CLEAN FINAL.docx]

**Epidemiology & Infection**

**Impacts of environmental factors on the aetiological diagnosis and disease severity of community-acquired pneumonia in China: A multicentre, hospital-based, observational study**

Yichunzi Zhang**,** Jiang Li**,** Chao Wu**,** Yan Xiao**,** Xinming Wang**,** Ying Wang**,** Lan Chen**,** Lili Ren and Jianwei Wang

Supplementary material

**Table of contents**

[Text S1. STROBE checklist 2](#_Toc164073149)

[Text S2. The criteria for case enrollment 5](#_Toc164073150)

[Text S3. Data collection and quality control 6](#_Toc164073151)

[Text S4. Models used for statistical analysis on variables with detected-pathogens and disease severity 7](#_Toc164073152)

[Table S1. The basic characteristics of nine hospitals during 2014-2019 8](#_Toc164073153)

[Table S2. Characteristics of community acquired pneumonia patients by pathogen detection 9](#_Toc164073154)

[Table S3. Characteristics of patients by severity of community-acquired pneumonia 11](#_Toc164073155)

[Table S4. Variance inflation factor of included variables in models for aetiological diagnosis and severity of disease 13](#_Toc164073156)

[Table S5. The distribution of missing value among study sample 14](#_Toc164073157)

[Table S6. Pathogen detection results rates in patients stratified by age, sex, and body mass index 15](#_Toc164073158)

[Table S7. Temperature, relative humidity, and pollutants characteristics of community-acquired pneumonia patients before admission and sampling 16](#_Toc164073159)

[Table S8. Adjusted ORs with 95% CIs for severe community-acquired pneumonia 17](#_Toc164073160)

[Table S9. Adjusted ORs with 95% CIs for overall and specific pathogen detection results in community-acquired pneumonia patients 18](#_Toc164073161)

[Figure S1. Association of environmental parametres with specific pathogens 19](#_Toc164073162)

[Figure S2. Association of specific environmental variables with the detection of specific pathogens and the diagnosis of severe community-acquired pneumonia (CAP) 19](#_Toc164073163)

[Figure S3. Association of specific environmental parametres with detection of overall pathogens according to distributed lag nonlinear models 19](#_Toc164073164)

[Figure S4. Association of environmental parametres with severe community-acquired pneumonia (CAP) in patients detected with specific pathogens 20](#_Toc164073165)

[Figure S5. Association of specific environmental parametres with severe community-acquired pneumonia according to distributed lag nonlinear models 20](#_Toc164073166)

[References 20](#_Toc164073167)

Text S1. STROBE checklist

|  | Item No | Recommendation | Location in manuscript | |  |
| --- | --- | --- | --- | --- | --- |
| **Title and abstract** | 1 | (*a*) Indicate the study’s design with a commonly used term in the title or the abstract | Title and Abstract | |  |
|  |  | (*b*) Provide in the abstract an informative and balanced summary of what was done and what was found | Abstract | |  |
| Introduction | | | |  | |
| Background/rationale | 2 | Explain the scientific background and rationale for the investigation being reported | Introduction | |  |
| Objectives | 3 | State specific objectives, including any prespecified hypotheses | Introduction | |  |
| Methods | | | |  | |
| Study design | 4 | Present key elements of study design early in the paper | Methods (Study design and population) | |  |
| Setting | 5 | Describe the setting, locations, and relevant dates, including periods of recruitment, exposure, follow-up, and data collection | Methods (Study design and population), Texts S2-S3, Table S1 | |  |
| Participants | 6 | (*a*) Give the eligibility criteria, and the sources and methods of selection of participants | Methods (Study design and population), Figure 1, Text S2, Table S1 | |  |
| Variables | 7 | Clearly define all outcomes, exposures, predictors, potential confounders, and effect modifiers. Give diagnostic criteria, if applicable | Methods (Study design and population, Procedures, Outcome measures, Statistical analysis), Texts S2-S4 | |  |
| Data sources/ measurement | 8* | For each variable of interest, give sources of data and details of methods of assessment (measurement). Describe comparability of assessment methods if there is more than one group | Methods (Procedures, Outcome measures, Statistical analysis), Texts S3-S4 | |  |
| Bias | 9 | Describe any efforts to address potential sources of bias | Methods (Study design and population, Procedures, Outcome measures, Statistical analysis), Texts S3-S4, Table S4 | |  |
| Study size | 10 | Explain how the study size was arrived at | Methods (Study design and population, Statistical analysis) | |  |
| Quantitative variables | 11 | Explain how quantitative variables were handled in the analyses. If applicable, describe which groupings were chosen and why | Methods (Procedures, Statistical analysis), Tables S2-S3 | |  |
| Statistical methods | 12 | (*a*) Describe all statistical methods, including those used to control for confounding | Methods (Statistical analysis), Text S4 | |  |
|  |  | (*b*) Describe any methods used to examine subgroups and interactions | Methods (Statistical analysis), Table S4 | |  |
|  |  | (*c*) Explain how missing data were addressed | Methods (Statistical analysis), Table S5 | |  |
|  |  | (*d*) If applicable, describe analytical methods taking account of sampling strategy | Methods (Statistical analysis) | |  |
|  |  | (*e*) Describe any sensitivity analyses | Methods (Statistical analysis) | |  |
| Results | | | |  | |
| Participants | 13* | (a) Report numbers of individuals at each stage of study—eg numbers potentially eligible, examined for eligibility, confirmed eligible, included in the study, completing follow-up, and analysed | Figure 1 | |  |
|  |  | (b) Give reasons for non-participation at each stage | Not applicable | |  |
|  |  | (c) Consider use of a flow diagram | Figure 1 | |  |
| Descriptive data | 14* | (a) Give characteristics of study participants (eg demographic, clinical, social) and information on exposures and potential confounders | Results, Text S2, Table 1, Tables S1-S3 and S6-S7 | |  |
|  |  | (b) Indicate number of participants with missing data for each variable of interest | Methods (Statistical analysis), Table 1, Tables S5-S6 | |  |
| Outcome data | 15* | Report numbers of outcome events or summary measures | Results, Table 1, Figure 2, Tables S2-S3 and S6-S7 | |  |
| Main results | 16 | (*a*) Give unadjusted estimates and, if applicable, confounder-adjusted estimates and their precision (eg, 95% confidence interval). Make clear which confounders were adjusted for and why they were included | Methods (Statistical analysis), Results, Figures 3-4, Tables S2-S3 and S8-S9, Figures S1-S5 | |  |
|  |  | (*b*) Report category boundaries when continuous variables were categorized | Methods (Procedures, Statistical analysis) | |  |
|  |  | (*c*) If relevant, consider translating estimates of relative risk into absolute risk for a meaningful time period | Not applicable | |  |
| Other analyses | 17 | Report other analyses done—eg analyses of subgroups and interactions, and sensitivity analyses | Results, Figures 3-4, Figures S2 and S4 | |  |
| Discussion | | | |  | |
| Key results | 18 | Summarise key results with reference to study objectives | Discussion | |  |
| Limitations | 19 | Discuss limitations of the study, taking into account sources of potential bias or imprecision. Discuss both direction and magnitude of any potential bias | Discussion | |  |
| Interpretation | 20 | Give a cautious overall interpretation of results considering objectives, limitations, multiplicity of analyses, results from similar studies, and other relevant evidence | Discussion | |  |
| Generalisability | 21 | Discuss the generalisability (external validity) of the study results | Discussion | |  |
| Other information | | | |  | |
| Funding | 22 | Give the source of funding and the role of the funders for the present study and, if applicable, for the original study on which the present article is based | Funding | |  |

Text S2. The criteria for case enrollment

**Inclusion and exclusion criteria of community-acquired pneumonia patients**

1. Inclusion criteria of community-acquired pneumonia patients

Community-acquired pneumonia (CAP) patients aged more than 14 years old were recruited when they have fever, cough, sore throat or other respiratory symptoms, and chest radiography confirmed pneumonia within 10 days post symptoms onset.

Severe CAP patients were defined as clinical CAP with ICU admission, or invasive mechanical ventilation, or septic shock with the need for vasopressors, or more than three of the following criteria [1]: ① respiratory rate≥30 breaths·min^−1^; ② PaO_2_/FiO_2_ ratio≦250; ③ multilobar infiltrates; ④ confusion/disorientation; ⑤ uraemia (BUN level≧20mg·dL^−1^); ⑥ leukopenia (WBC count < 4000 cells·mm^−3^); ⑦ thrombocytopenia (platelet count < 100000 cells·mm^−3^); ⑧ hypothermia (core temperature <36°C); ⑨ hypotension requiring aggressive fluid resuscitation.

2. Exclusion criteria of community-acquired pneumonia patients included (1) inability to obtain informed consent; (2) immunosuppression or tolerance, immune function-related gene defects, including but not limited to malignant tumor, organ transplantation, human immunodeficiency virus infection, and use of immunosuppressants for 30 consecutive days before onset; and (3) alternative diagnoses, including pulmonary tuberculosis, pulmonary tumor, noninfectious pulmonary interstitial disease, pulmonary oedema, atelectasis, pulmonary embolism, pulmonary eosinophilic infiltration and pulmonary vasculitis.

Text S3. Data collection and quality control

Trained physicians in the hospitals were responsible for sampling according to the standard operational procedure. All the samples were sent to the central laboratory and tested by the fixed operators using the same kits (Fast Track Diagnostics, Junglinster Luxembourg) and protocols. A total of 33 common respiratory pathogens were screened, including influenza A, B, and C (IFVs); human parainfluenza viruses 1, 2, 3 and 4 (HPIVs); human coronaviruses NL63, 229E, OC43, and HKU1 (HCoVs); human metapneumoviruses A and B (HMPVs); human rhinovirus (HRV); respiratory syncytial viruses A and B (RSVs); human adenovirus (HAdv); enterovirus (EV); parechovirus; human bocavirus (HBoV); cytomegalovirus (CMV); *Pneumocystis jiroveci* (*P. jirovecii*); *Mycoplasma pneumoniae* (*M. pneumoniae*); *Chlamydia pneumoniae* (*C. pneumoniae*); *Streptococcus pneumoniae* (*S. pneumoniae*); *Haemophilus influenzae type B* (*H. influenzae*); *Haemophilus parahaemolyticus* (*H. parahaemolyticus*); *Staphylococcus aureus* (*S. aureus*); *Moraxella catarrhalis* (*M. catarrhalis*); *Bordetella* spp. (except *Bordetella parapertussis*); *Klebsiella pneumoniae* (*K. pneumoniae*); *Legionella* spp.; and *Salmonella* spp. Negative and positive-controls were included to exclude potential contamination and confirm the quality of the amplification. The demographic data, physical examination, diagnosis, clinical treatment information was obtained from clinical record. All data was backed up.

Text S4. Models used for statistical analysis on variables with detected-pathogens and disease severity

1. The model of the distributed lag nonlinear model (DLNM) for pathogen detection results is as follows:

Logit[*E*(*Y_it_*)] = *α* + *β*X*_it_*_,_ *_l_* + *ns*(Tem*_it_*, 3df) + *ns*(Time*_it_*, 7df/year) + factor(Dow*_it_*) + Age*_i_* + factor(sexy*_i_*) + factor(BMI*_i_*) + factor(AP*_i_*) + TFSOA*_i_* + factor(PSI*_i_*) + factor(area*_i_*) In the model above, *t* represents the day of observation, and *l* is the lag days. *Y_it_* denotes the detection result of patient *i*, *α* is the constant term, X*_it_*_,_ *_l_* refers to the cross-basis matrix obtained by applying the DLNM to six air pollutants (PM2.5, PM10, SO_2_, NO_2_, O_3_-8h, and CO) and RH, and *β* refers to the vector of coefficients for X*_it_*_,_ *_l_*. A cross-basis function was developed to analyze each environmental parametre, and natural cubic spline function with 3 degrees of freedom (df) was used to model the lag-response relationship. A linear function was used to fit the exposure-response relationship between each environmental parametre and detected pathogen. *ns*(Tem*_it_*, 3df) is the natural cubic spline functions with 3 df to account for the potential nonlinear eﬀects of temperature averaged over the 3 days before and contain day *t*. *ns*(Time*_it_*, 7df/year) is a natural cubic spline function of time with 7 df per year to capture both the long-term trend and seasonality. Dow*_it_* indicates the day of the week. PSI was adjusted to consider the potential effect on detected results by severity. The area means the city of hospital. For *K. pneumoniae*, X*_it_*_,_ *_l_* refers to the cross-basis matrix to SO_2_ or O_3_-8h. After averaging over the 7 days before and contain day *t*, other environmental parametres were the natural cubic spline functions with 3 df.

2. The model of DLNM for severity of CAP is as follows:

Logit[*E*(*Y_it_*)] = *α* + *β*X*_it_*_,_ *_l_* + *ns*(Tem*_it_*, 3df) + *ns*(Time*_it_*, 7df/year) + factor(Dow*_it_*) + Age*_i_* + factor(sexy*_i_*) + factor(BMI*_i_*) + factor(AP*_i_*) + TFSOA*_i_* + factor(Pathogen*_i_*) + factor(area*_i_*) In the model above, *Y_it_* denotes the severity result of patient *i*. A linear function was used to fit the exposure-response relationship between each environmental parametre and severity of disease. Pathogen results was coded as binary variables, which was adjusted to consider the potential effect on severity by detected results. The explanations for the other parametres are the same as in the previous equation.

Table S1. The basic characteristics of nine hospitals during 2014-2019

| **City** | **Region** | **Province** | **Hospital name** |
| --- | --- | --- | --- |
| Harbin | Northeast China | Heilongjiang | The Second Affiliated Hospital of Harbin Medical University |
| Changchun | Northeast China | Jilin | Bethune First Hospital of Jilin University |
| Xi’an | Northwest China | Shanxi | Xijin Hospital of the Fourth Military Medical University |
| Xi’an | Northwest China | Shanxi | The First Affiliated Hospital of Xi’an Jiaotong University |
| Wuhan | Central China | Hubei | Tongji Hospital of Tongji Medical College of HUST |
| Chengdu | Southwest China | Sichuan | West China Hospital of Sichuan University |
| Nanjing | East China | Jiangsu | Zhongda Hospital of Southeast University |
| Fuzhou | East China | Fujian | Fujian Provincial Hospital |
| Shenzhen | South China | Guangdong | The Third People’s Hospital of Shenzhen |

*P* value for missing value using chi-square or fisher’s exact test (if cell number <5)

Table S2. Characteristics of community acquired pneumonia patients by pathogen detection

| **Variables** | **Nonpositive detection (n=1259)** | **Bacteria (fungus) (n=942)** | **Viruses (n=653)** | **Multiple pathogens (n=469)** | ***P* value** |
| --- | --- | --- | --- | --- | --- |
| Age, years; median (IQR) | 59 (25) | 52 (35) | 61 (25) | 59 (32) | <0.001^a^ |
| Sex |  |  |  |  |  |
| Female; n (%) | 452 (35.9) | 378 (40.1) | 247 (37.8) | 174 (37.1) | 0.102^b^ |
| Male; n (%) | 767 (60.9) | 513 (54.5) | 382 (58.5) | 274 (58.4) |  |
| BMI, kg/m^2^ |  |  |  |  |  |
| <25; n (%) | 989 (78.6) | 767 (81.4) | 488 (74.7) | 365 (77.8) | 0.046^b^ |
| ≥25; n (%) | 210 (16.7) | 134 (14.2) | 125 (19.1) | 81 (17.3) |  |
| BS, variables |  |  |  |  |  |
| Temperature, °C; median (IQR) | 15.8 (16.0) | 19.6 (12.8) | 14.3 (14.9) | 15.9 (17.2) | <0.001^a^ |
| RH, %; median (IQR) | 65.7 (21.5) | 71.1 (19.5) | 70.9 (21.7) | 70.4 (19.7) | <0.001^a^ |
| PM2.5, μg/m^3^; median (IQR) | 46.1 (38.6) | 38.3 (31.7) | 46.0 (40.0) | 41.1 (35.6) | <0.001^a^ |
| PM10, μg/m^3^; median (IQR) | 82.2 (71.9) | 67.3 (52.0) | 81.7 (62.0) | 72.9 (57.5) | <0.001^a^ |
| SO_2_, μg/m^3^; median (IQR) | 13.0 (14.9) | 9.8 (8.8) | 12.2 (12.3) | 11.9 (11.4) | <0.001^a^ |
| NO_2_, μg/m^3^; median (IQR) | 45.2 (22.2) | 37.4 (20.4) | 44.5 (22.2) | 41.6 (22.1) | <0.001^a^ |
| O_3_-8h, μg/m^3^; median (IQR) | 50.2 (34.5) | 54.4 (33.0) | 46.9 (33.2) | 50.0 (32.3) | <0.001^a^ |
| CO, mg/m^3^; median (IQR) | 1.0 (0.5) | 0.9 (0.4) | 1.0 (0.4) | 0.9 (0.5) | <0.001^a^ |
| AP |  |  |  |  |  |
| No; n (%) | 1018 (80.9) | 708 (75.2) | 474 (72.6) | 341 (72.7) | <0.001^b^ |
| Yes; n (%) | 241 (19.1) | 234 (24.8) | 179 (27.4) | 128 (27.3) |  |
| PSI score |  |  |  |  |  |
| <90; n (%) | 1059 (84.1) | 789 (83.8) | 501 (76.7) | 371 (79.1) | <0.001^b^ |
| ≥90; n (%) | 200 (15.9) | 153 (16.2) | 152 (23.3) | 98 (20.9) |  |
| TFSOA, days; median (IQR) | 7 (6) | 6 (5) | 7 (7) | 6 (5) | <0.001^a^ |

Except sex and BMI, not all percentages add up to 100% due to rounding. IQR=interquartile range. BMI=body mass index. BS=before sampling. RH=relative humidity. PM=particulate matter. SO_2_=sulfur dioxide. NO_2_=nitrogen dioxide. O_3_-8h=8-hour ozone levels. CO=carbon monoxide. AP=antibiotics pre-admission. PSI=pneumonia severity index. TFSOA=time from symptom onset to admission.

a Kruskal-Wallis H test

b χ^2^ test

Table S3. Characteristics of patients by severity of community-acquired pneumonia

| **Variables** | **Severe community-acquired pneumonia (n=709)** | **Nonsevere Community-acquired pneumonia (n=2614)** | ***P* value** |
| --- | --- | --- | --- |
| Age, years; median (IQR) | 63 (25) | 56 (31) | <0.001^a^ |
| Sex |  |  |  |
| Female; n (%) | 192 (27.1) | 1059 (40.5) | <0.001^b^ |
| Male; n (%) | 496 (70.0) | 1440 (55.1) |  |
| BMI, kg/m^2^ |  |  |  |
| <25; n (%) | 531 (74.9) | 2078 (79.5) | 0.777^b^ |
| ≥25; n (%) | 109 (15.4) | 441 (16.9) |  |
| Pathogen |  |  |  |
| Nonpositive detection; n (%) | 236 (33.3) | 1023 (39.1) | <0.001^b^ |
| Bacteria (fungus); n (%) | 195 (27.5) | 747 (28.6) |  |
| Viruses; n (%) | 174 (24.5) | 479 (18.3) |  |
| Multiple pathogens; n (%) | 104 (14.7) | 365 (14.0) |  |
| BA, variables |  |  |  |
| Temperature, °C; median (IQR) | 15.7 (14.2) | 17.0 (15.6) | 0.009^a^ |
| RH, %; median (IQR) | 67.3 (19.1) | 69.4 (21.9) | 0.010^a^ |
| PM2.5, μg/m^3^; median (IQR) | 43.7 (33.1) | 42.3 (36.2) | 0.072^a^ |
| PM10, μg/m^3^; median (IQR) | 80.3 (62.0) | 75.3 (62.1) | 0.011^a^ |
| SO_2_, μg/m^3^; median (IQR) | 12.2 (11.7) | 11.5 (11.8) | 0.124^a^ |
| NO_2_, μg/m^3^; median (IQR) | 43.5 (20.6) | 42.5 (23.2) | 0.302^a^ |
| O_3_-8h, μg/m^3^; median (IQR) | 53.0 (34.0) | 50.5 (33.7) | 0.596^a^ |
| CO, mg/m^3^; median (IQR) | 1.0 (0.4) | 1.0 (0.5) | 0.848^a^ |
| AP |  |  |  |
| No; n (%) | 494 (69.7) | 2047 (78.3) | <0.001^b^ |
| Yes; n (%) | 215 (30.3) | 567 (21.7) |  |
| TFSOA, days; median (IQR) | 7 (6) | 7 (7) | 0.318^a^ |

Except sex and BMI, not all percentages add up to 100% due to rounding. IQR=interquartile range. BMI=body mass index. BA=before admission. RH=relative humidity. PM=particulate matter. SO_2_=sulfur dioxide. NO_2_=nitrogen dioxide. O_3_-8h=8-hour ozone levels. CO=carbon monoxide. AP=antibiotics pre-admission. TFSOA=time from symptom onset to admission.

a Mann-Whitney U test

b χ^2^ test

Table S4. Variance inflation factor of included variables in models for aetiological diagnosis and severity of disease

| **Variables** | **Overall pathogens** | **severe CAP** |
| --- | --- | --- |
| Age, years | 1.274 | 1.049 |
| Sex | 1.047 | 1.024 |
| Body mass index, kg/m^2^ | 1.024 | 1.024 |
| Temperature, °C | 3.364 | 3.326 |
| Relative humidity, 10 % | 1.518 | 1.498 |
| Particulate matter 2.5, 10 μg/m^3^ | 7.475 | 7.506 |
| Particulate matter 10, 10 μg/m^3^ | 7.996 | 8.050 |
| Sulfur dioxide, 10 μg/m^3^ | 2.527 | 2.510 |
| Nitrogen dioxide, 10 μg/m^3^ | 3.035 | 3.062 |
| 8-hour ozone levels, 10 μg/m^3^ | 1.916 | 1.887 |
| Carbon monoxide, 1 mg/m^3^ | 2.063 | 2.083 |
| Antibiotics pre-admission | 1.044 | 1.038 |
| Time from symptom onset to admission, days | 1.062 | 1.070 |
| Pneumonia severity index score | 1.278 | - |
| Pathogen | - | 1.076 |

Table S5. The distribution of missing value among study sample

| **Variables** | **Nonpositive detection (n=1259)** | **Positive pathogen**  **(n=2064)** | ***P* value** | **Severe pneumonia**  **(n=709)** | **Nonsevere pneumonia**  **(n=2614)** | ***P* value** |
| --- | --- | --- | --- | --- | --- | --- |
| Age |  |  |  |  |  |  |
| Non-missing | 1122 | 1804 | 0.139 | 661 | 2265 | <0.001 |
| Missing | 137 | 260 |  | 48 | 349 |  |
| Sex |  |  |  |  |  |  |
| Non-missing | 1219 | 1968 | 0.037 | 688 | 2499 | 0.087 |
| Missing | 40 | 96 |  | 21 | 115 |  |
| Body mass index |  |  |  |  |  |  |
| Non-missing | 1199 | 1960 | 0.724 | 640 | 2519 | <0.001 |
| Missing | 60 | 104 |  | 69 | 95 |  |

Table S6. Pathogen detection results rates in patients stratified by age, sex, and body mass index

| **Variables** | **<60 years**  **(n=1543)** | **≥60 years**  **(n=1383)** | **Female**  **(n=1251)** | **Male**  **(n=1936)** | **BMI<25 kg/m^2^**  **(n=2609)** | **BMI≥25 kg/m^2^**  **(n=550)** |
| --- | --- | --- | --- | --- | --- | --- |
| Nonpositive detection, n (%) | 562 (36.4) | 560 (40.5) | 452 (36.1) | 767 (39.6) | 989 (37.9) | 210 (38.2) |
| Bacteria (fungus), n (%) | 500 (32.4) | 317 (22.9) | 378 (30.2) | 513 (26.5) | 767 (29.4) | 134 (24.4) |
| Viruses, n (%) | 267 (17.3) | 306 (22.1) | 247 (19.7) | 382 (19.7) | 488 (18.7) | 125 (22.7) |
| Multiple pathogens, n (%) | 214 (13.9) | 200 (14.5) | 174 (13.9) | 274 (14.2) | 365 (14.0) | 81 (14.7) |

Not all percentages add up to 100% due to rounding. BMI=body mass index.

Table S7. Temperature, relative humidity, and pollutants characteristics of community-acquired pneumonia patients before admission and sampling

| **Variables** | **Before admission**  **(n=3323)** | **Before sampling**  **(n=3323)** |
| --- | --- | --- |
| Temperature, °C; median (IQR) | 16.5 (15.4) | 16.5 (15.4) |
| RH, %; median (IQR) | 68.9 (21.3) | 69.0 (21.1) |
| PM2.5, μg/m^3^; median (IQR) | 42.7 (35.6) | 42.6 (35.6) |
| PM10, μg/m^3^; median (IQR) | 76.3 (61.6) | 76.2 (61.5) |
| SO_2_, μg/m^3^; median (IQR) | 11.7 (11.8) | 11.7 (11.7) |
| NO_2_, μg/m^3^; median (IQR) | 42.7 (22.6) | 42.6 (22.6) |
| O_3_-8h, μg/m^3^; median (IQR) | 51.0 (33.6) | 51.1 (33.5) |
| CO, mg/m^3^; median (IQR) | 1.0 (0.5) | 1.0 (0.5) |

IQR=interquartile range. RH=relative humidity. PM=particulate matter. SO_2_=sulfur dioxide. NO_2_=nitrogen dioxide. O_3_-8h=8-hour ozone levels. CO=carbon monoxide.

Table S8. Adjusted ORs with 95% CIs for severe community-acquired pneumonia

|  | **Adjusted ORs (95% CIs)** |
| --- | --- |
| Age, years | 1.09 (1.07-1.12) * |
| Sex |  |
| Female | 1 (ref) |
| Male | 1.83 (1.51-2.21) * |
| BMI, kg/m^2^ |  |
| <25 | 1 (ref) |
| ≥25 | 0.87 (0.68-1.10) |
| Pathogen |  |
| Nonpositive detection | 1 (ref) |
| Positive detection | 1.40 (1.16-1.68) * |
| TFSOA, days | 1.01 (0.99-1.03) |

ORs for severe community-acquired pneumonia, adjusted for age, sex, BMI, pathogen, temperature, parametres with lag 0-6 days (relative humidity, PM2.5, PM10, sulfur dioxide, nitrogen dioxide, 8-hour ozone levels, carbon monoxide), antibiotics pre-admission, TFSOA, area, and admission time. OR=odds ratio. BMI=body mass index. PM=particulate matter. TFSOA=time from symptom onset to admission. **P*<0.05.

Table S9. Adjusted ORs with 95% CIs for overall and specific pathogen detection results in community-acquired pneumonia patients

|  | **Adjusted ORs (95% CIs)** | | | | | | |
| --- | --- | --- | --- | --- | --- | --- | --- |
|  | **Overall pathogens** | ***M. pneumoniae*** | ***H. influenzae*** | ***K. pneumoniae*** | ***S. pneumoniae*** | **IFVs** | **HRV** |
| Age, years | 0.96 (0.94-0.99) * | 0.83 (0.80-0.86) * | 1.02 (0.99-1.06) | 1.06 (1.02-1.10) * | 1.04 (1.00-1.08) | 1.04 (1.00-1.08) * | 1.04 (1.00-1.08) |
| Sex |  |  |  |  |  |  |  |
| Female | 1 (ref) | 1 (ref) | 1 (ref) | 1 (ref) | 1 (ref) | 1 (ref) | 1 (ref) |
| Male | 0.84 (0.72-0.98) * | 0.61 (0.48-0.77) * | 0.91 (0.71-1.16) | 1.37 (1.06-1.77) * | 1.55 (1.16-2.08) * | 0.92 (0.73-1.17) | 1.00 (0.79-1.28) |
| BMI, kg/m^2^ |  |  |  |  |  |  |  |
| <25 | 1 (ref) | 1 (ref) | 1 (ref) | 1 (ref) | 1 (ref) | 1 (ref) | 1 (ref) |
| ≥25 | 1.00 (0.82-1.23) | 0.92 (0.67-1.27) | 0.94 (0.69-1.30) | 0.88 (0.64-1.21) | 0.86 (0.60-1.24) | 1.39 (1.05-1.83) * | 0.94 (0.68-1.29) |
| AP |  |  |  |  |  |  |  |
| No | 1 (ref) | 1 (ref) | 1 (ref) | 1 (ref) | 1 (ref) | 1 (ref) | 1 (ref) |
| Yes | 1.45 (1.21-1.73) * | 1.75 (1.36-2.25) * | 0.99 (0.75-1.31) | 1.15 (0.88-1.50) | 1.10 (0.81-1.50) | 1.46 (1.13-1.88) * | 0.81 (0.61-1.09) |
| TFSOA, days | 0.96 (0.95-0.98) * | 0.94 (0.91-0.96) * | 0.96 (0.93-0.99) * | 0.98 (0.95-1.00) | 0.93 (0.90-0.97) * | 0.96 (0.93-0.98) * | 1.02 (1.00-1.05) |

ORs for overall pathogen result and specific results (*Mycoplasma pneumoniae*, *Haemophilus influenzae*, *Klebsiella pneumoniae*, *Streptococcus pneumoniae*, influenza viruses, and human rhinovirus) of CAP. Adjusted for age, sex, BMI, temperature, parametres with lag 0-6 days (relative humidity, PM2.5, PM10, sulfur dioxide, nitrogen dioxide, 8-hour ozone levels, carbon monoxide), AP, TFSOA, pneumonia severity index score, area, and admission time. OR=odds ratio. BMI=body mass index. PM=particulate matter. AP=antibiotics pre-admission. TFSOA=time from symptom onset to admission. **P*<0.05

Figure S1. Association of environmental parametres with specific pathogens

Adjusted ORs for *Mycoplasma pneumoniae*, *Haemophilus influenzae*, *Streptococcus pneumoniae*, and human rhinovirus of community-acquired pneumonia after adjusting for age, sex, body mass index, temperature, RH, PM2.5, PM10, SO_2_, NO_2_, O_3_-8h, CO, antibiotics pre-admission, time from symptom onset to admission, pneumonia severity index score, area, and admission time. OR=odds ratio. RH=relative humidity. PM=particulate matter. SO_2_=sulfur dioxide. NO_2_=nitrogen dioxide. O_3_-8h=8-hour ozone levels. CO=carbon monoxide

Figure S2. Association of specific environmental variables with the detection of specific pathogens and the diagnosis of severe community-acquired pneumonia (CAP)

a. For association of PM2.5 on detection of *Haemophilus influenzae*, exposure-response curve at lag 0 day according to distributed lag nonlinear model (DLNM), and single-exposure effects (95% CI) according to bayesian kernel machine regression (BKMR). The dashed line in DLNM is 75 μg/m^3^, representing the concentration of emission standard. b. Exposure-response curve at lag 4 days for association of SO_2_ on detection of *Klebsiella pneumoniae*, and exposure-response curve at lag 2 days for association of NO_2_ on detection of human rhinovirus. The dashed lines represent half of the emission standard (75 μg/m^3^) of SO_2_, and minimum of NO_2_ (10 μg/m^3^). c. Exposure-response curve at lag 4 days for association of relative humidity on detection of viruses. The dashed line represents 50%. d. Single-exposure effects for association of pollutants on severe CAP in patients detected with human rhinovirus. Effects from BKMR were defined as the change in the response associated with a change in a particular exposure from its 25th to its 75th percentile, where all of the other exposures are fixed at a specific quantile (0.25, 0.50, or 0.75). OR=odds ratio. PM=particulate matter. SO_2_=sulfur dioxide. NO_2_=nitrogen dioxide. O_3_-8h=8-hour ozone levels. CO=carbon monoxide.

Figure S3. Association of specific environmental parametres with detection of overall pathogens according to distributed lag nonlinear models

The dashed line represents the emission standard concentration except 8-hour ozone levels and relative humidity, which is 75 μg/m^3^ for particulate matter 2.5, 150 μg/m^3^ for particulate matter 10 and sulfur dioxide, 80 μg/m^3^ for nitrogen dioxide, 4 mg/m^3^ for carbon monoxide, half of emission standard concentration (80 μg/m^3^) for 8-hour ozone levels, and 50% for relative humidity. OR=odds ratio.

Figure S4. Association of environmental parametres with severe community-acquired pneumonia (CAP) in patients detected with specific pathogens

Adjusted ORs for severe CAP in CAP patients detected with *Klebsiella pneumoniae*, *Haemophilus influenzae*, *Streptococcus pneumoniae*, influenza viruses, and human rhinovirus. Adjusted for age, sex, body mass index, temperature, RH, PM2.5, PM10, SO_2_, NO_2_, O_3_-8h, CO, antibiotics pre-admission, time from symptom onset to admission, area, and admission time. OR=odds ratio. RH=relative humidity. PM=particulate matter. SO_2_=sulfur dioxide. NO_2_=nitrogen dioxide. O_3_-8h=8-hour ozone levels. CO=carbon monoxide.

Figure S5. Association of specific environmental parametres with severe community-acquired pneumonia according to distributed lag nonlinear models

The dashed line represents the emission standard concentration except 8-hour ozone levels and relative humidity, which is 75 μg/m^3^ for particulate matter 2.5, 150 μg/m^3^ for sulfur dioxide, 80 μg/m^3^ for nitrogen dioxide, half of emission standard concentration (80 μg/m^3^) for 8-hour ozone levels, and 50% for relative humidity. OR=odds ratio.

References

[1] Mandell LA, et al. (2007) Infectious Diseases Society of America/American Thoracic Society consensus guidelines on the management of community-acquired pneumonia in adults. *Clinical infectious diseases*;**44 Suppl 2**:S27-S72.
